# Updated Functional Roles of NAMPT in Carcinogenesis and Therapeutic Niches

**DOI:** 10.3390/cancers14092059

**Published:** 2022-04-19

**Authors:** Tsung-Chieh Lin

**Affiliations:** Genomic Medicine Core Laboratory, Department of Medical Research and Development, Chang Gung Memorial Hospital, New Taipei City 333, Taiwan; tclin1980@cgmh.org.tw; Tel.: +886-3-3281200 (ext. 7722)

**Keywords:** NAMPT, prognosis, cancer progression

## Abstract

**Simple Summary:**

The advantages and applications of using the non-invasive way to detect serum biomarkers for assessing cancer diagnosis and prognosis have been explored. Nicotinamide phosphoribosyltransferase (NAMPT), also designated as pre-B-cell colony-enhancing factor (PBEF) or visfatin, is a secreted adipokine known to modulate tumor malignancies. Its significance in predicting cancer patient’s survival outcome further renders the implementation of NAMPT in clinical practice. In this review, recent discoveries of NAMPT in cancer studies were focused and integrated. We aim to provide updates for researchers who are proposing relevant objectives.

**Abstract:**

Nicotinamide phosphoribosyltransferase (NAMPT) is notable for its regulatory roles in tumor development and progression. Emerging evidence regarding NAMPT somatic mutations in cancer patients, NAMPT expressional signatures in normal tissues and cancers, and the prognostic significance of NAMPT in many cancer types has attracted attention, and NAMPT is considered a potential biomarker of cancer. Recent discoveries have demonstrated the indirect association and direct biological functions of NAMPT in modulating cancer metastasis, proliferation, angiogenesis, cancer stemness, and chemoresistance to anticancer drugs. These findings warrant further investigation of the underlying mechanisms to provide knowledge for developing novel cancer therapeutics. In this review article, we explore recent research developments involving the oncogenic activities of NAMPT by summarizing current knowledge regarding NAMPT somatic mutations, clinical trials, transcriptome data, and clinical information and discoveries related to the NAMPT-induced signaling pathway in modulating hallmarks of cancer. Furthermore, the comprehensive representation of NAMPT RNA expression in a pancancer panel as well as in specific normal cell types at single-cell level are demonstrated. The results suggest potential sites and cell types that could facilitate NAMPT-related tumorigenesis. With this review, we aim to shed light on the regulatory roles of NAMPT in tumor development and progression, and provide information to guide future research directions in this field.

## 1. Introduction

**Figure 1:**
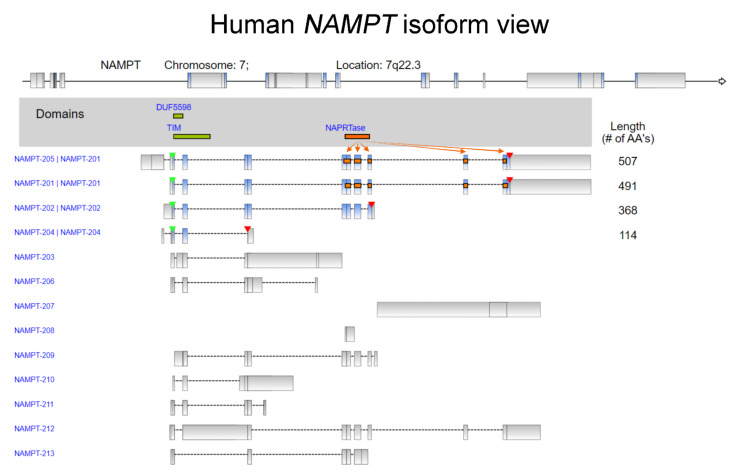
Isoform view of human *NAMPT*. The position of the stop codon and start site of transcription are indicated by red and green arrowheads, respectively. The matched NAPRTase protein domain in each isoform is marked in orange. The data were retrieved from Ensembl and analyzed.

Nicotinamide phosphoribosyltransferase (NAMPT) is a pivotal NAD biosynthetic enzyme and critical participant in the modulation of cell metabolic activities, reprogramming, senescence, and cell apoptosis [1]. It is also known as adipocytokine visfatin or pre-B-cell colony-enhancing factor (PBEF), which has been characterized as a secreted protein from peripheral blood lymphocytes in humans [2]. NAMPT plays a critical role in the enzymatic process of nicotinamide adenine dinucleotide NAD^+^ salvage pathway [3,4]. Extracellular NAMPT (eNAMPT) has been uncovered to play a role as an inflammatory cytokine that is independent of the catalytic activity shown by the intracellular form, iNAMPT [5]. In addition, eNAMPT in conditioned medium could synergize the effects of stem cell factor and interleukin 7 that the co-stimulation of NAMPT increased the number of pre-B-cell colony formation [2]. To date, accumulating results indicate that the human NAMPT gene encodes mRNA that is spliced into a total of 13 transcript variants varying from length, stop codon, transcriptional start site, and protein domain (Figure 1). Among those variants, NAPRTase domain was solely discovered in NAMPT-205 and NAMPT-201, and the matched locations were aligned and marked in orange (Figure 1). Importantly, the abnormal NAMPT expression level in various types of cancers has been detected. The statistically significant association between the circulating NAMPT (visfatin) levels and the cancer risk has been discovered [6]. In addition, emerging evidence has indicated the NAMPT expression in a broad range of tumor types and the NAMPT-mediated biological impacts modulating several critical processes in tumor development, including induction of angiogenesis, cancer cell proliferation, cancer metastasis, and chemoresistance. In this review article, we provide an overview of the up-to-date knowledge regarding NAMPT-mediated biological effects across cancers. NAMPT expression in normal tissues is first presented at the single-cell level. We integrated and summarized the current knowledge on this topic, focusing on *NAMPT* somatic mutations and evidence of NAMPT expression in different tumor types and on NAMPT-mediated biological functions in modulating cancer progression. Furthermore, the prognostic significance of NAMPT is illustrated in a pancancer panel.

## 2. NAMPT Somatic Mutations and Cancer

**Figure 2:**
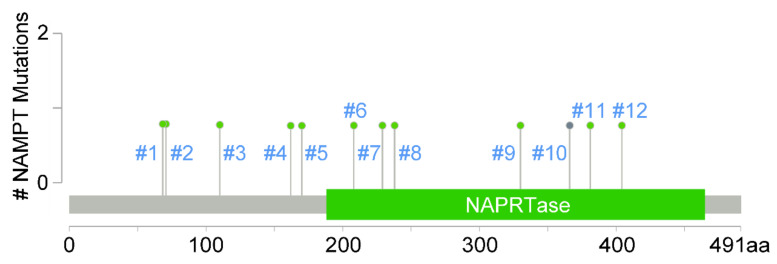
A pancancer study of whole genomes revealing types and sites of *NAMPT* gene mutations. Mutation diagram circles are highlighted by colors with respect to the specific mutation types. Light green indicates missense mutations (unknown significance). Gray indicates truncating mutations (putative driver), including frameshift deletion, nonsense, nonstop, and frameshift insertion mutations.

Targeting NAMPT has been proposed as an anticancer strategy, and several NAMPT-selective inhibitors have been uncovered to significantly block NAMPT’s function in tumor cells as well as the attenuation of cancer progression. However, the occurrence of chemoresistance commonly limits the development of tumor therapeutics. An exome sequencing study of the NAMPT gene in cells resistant to inhibitor revealed a heterozygous point mutation which did not exist in the parental cell line [7]. Several polymorphisms were identified in *NAMPT* in a study screening normal and tumor samples from different tissues and populations [8]. In addition, mutation of residue Ser165 was confirmed to elicit the unwinding of an α-helix which interacts with the NAMPT downstream substrate, 5-phosphoribosyl-1-pyrophosphate (PRPP). Mutation of the Gly217 residue led to the orthosteric blocking of inhibitor binding [9]. Moreover, a pancancer analysis of whole-genome data that integrated 2658 whole-cancer genomes and matched normal tissues across 38 tumor types from the International Cancer Genome Consortium (ICGC) and The Cancer Genome Atlas (TCGA) was recently published [10]. *NAMPT* mutation signatures in pancancer types were retrieved and summarized from the public database cBioPortal (https://www.cbioportal.org/, accessed on 28 February 2022) [11,12] and are shown in Figure 2 and Table 1.

## 3. NAMPT Distribution and Expression in Normal Cell Types

**Figure 3:**
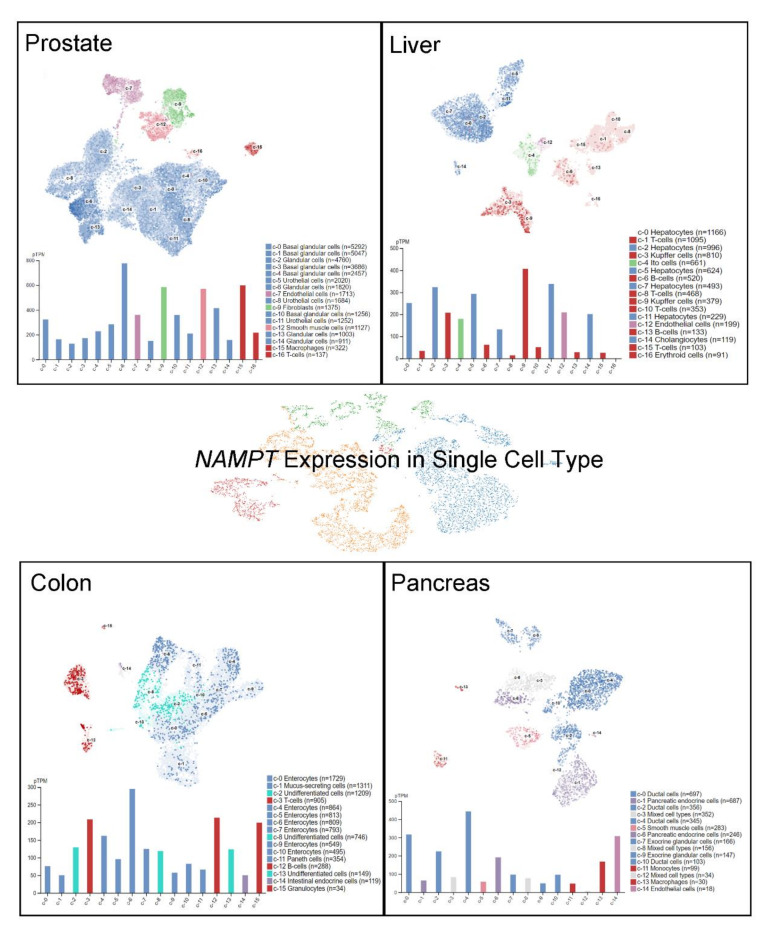
Human *NAMPT* expression in single cells of different cell types. The *NAMPT* level was detected by scRNA-seq in various tissues. The RNA expression levels in the cell type clusters were identified in each tissue and visualized by using the UMAP plot of single cells.

**Figure 4:**
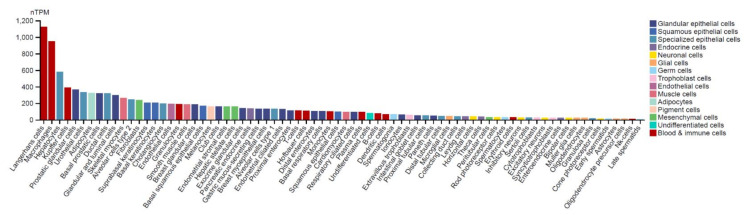
NAMPT RNA levels were measured by scRNA-seq in 192 specific cell type clusters, and the relative expression levels are presented. nTPM: TPM values of all samples were normalized separately using the trimmed mean of M values (TMM) to allow for between-sample comparisons and normalized transcript expression values.

To delineate the different and specific cell types in a given tissue, *NAMPT* RNA distribution on a panorgan scale has been investigated by single-cell RNA sequencing (scRNA-seq) [13,14,15,16]. Notably, the understanding of single-cell RNA expression in specific cell types in normal tissues might shed light on the further exploration of specific cancer biomarkers that are potentially critical to the development of cancer [17]. A recently published cell-type atlas shows the scRNA-seq data in 192 specific clusters of cell types (the Human Protein Atlas, https://www.proteinatlas.org/ accessed on 28 February 2022) [18]. *NAMPT* expression levels in normal organs, namely, the prostate, liver, colon, and pancreas, are shown at the single-cell level in Figure 3. Relatively higher *NAMPT* expression has been observed in glandular cells, fibroblasts, smooth muscle cells and macrophages in the prostate. Hepatocytes, Kupffer cells, Ito cells, endothelial cells and cholangiocytes in the liver all have been reported to exhibit significant *NAMPT* expression. In addition, *NAMPT* expression in the rectum has been specifically detected in T cells, enterocytes, B cells, and granulocytes, but not in other cell types, suggesting the potential stemness of specific *NAMPT*-expressing cells. In pancreatic tissues, NAMPT RNA expression has been found in ductal cells, pancreatic endocrine cells, macrophages, and endothelial cells. These findings further indicate the potential sites of NAMPT-dependent effects that may participate in tumorigenesis. Moreover, an overall illustration of *NAMPT* RNA expression levels across completely normal cell types is presented, demonstrating the top 5 specific cell types displaying high NAMPT levels: Langerhans cells, macrophages, hepatocytes, Kupffer cells, and prostatic glandular cells (Figure 4).

## 4. NAMPT Expression Levels in Cancers

**Figure 5:**
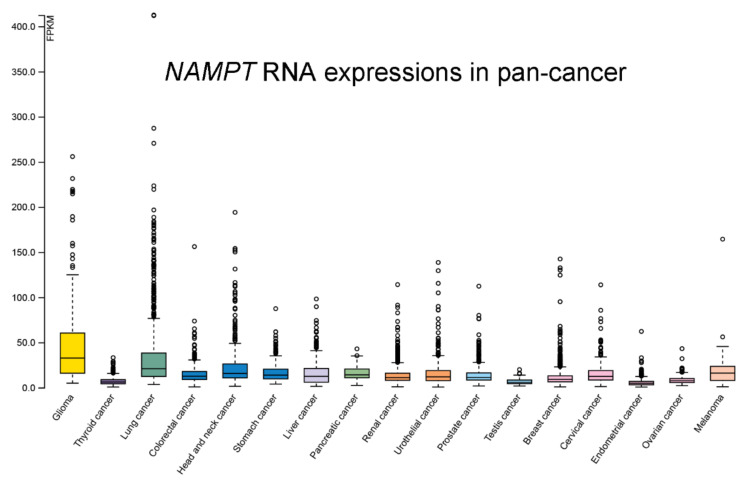
*NAMPT* RNA-seq data for 17 cancer types from TCGA were analyzed and reported as the median number of fragments per kilobase of exon per million reads (FPKM). Normal distribution across the dataset is represented by box plots, shown as the median, 25^th^, and 75th percentiles. The points represented the data of outliers if the expression levels are below or above 1.5 times the interquartile range. The data for the analyses were retrieved from HPA database (https://www.proteinatlas.org/, accessed on 28 February 2022).

NAMPT has been detected in both plasma and tumors from cancer patients, suggesting the potential diagnostic significance of NAMPT in cancer. The results of luciferase reporter assays and bioinformatics analysis both characterized the NAMPT 3’-untranslated region as a direct target of miR-613 [19]. Robust increase of NAMPT was reported in the tissue biopsies of MM patients diagnosed with the occurrence of chemoresistance to BRAFi (*p* < 0.001) [20]. Serum NAMPT (visfatin) concentrations were increased in a cohort of 69 hepatocellular carcinoma patients compared to control subjects [21]. Similarly, the serum levels of NAMPT and miR-21 were significantly higher in hepatocellular carcinoma patients than in healthy subjects [22]. An analysis of public data extracted from the Oncomine and PrognoScan databases indicated the overexpression of NAMPT in human colorectal cancer [23]. Furthermore, higher NAMPT levels were detected in colorectal cancer tissues than in paired normal tissues, especially in cancer patients with stage I and II disease [24]. The data of an analysis using tissue array from oral squamous cell carcinoma indicated the upregulation of NAMPT in cancer [25]. Furthermore, increased levels of NAMPT were uncovered in tumor tissues in the study of 8 NT-matched pairs of pancreatic ductal adenocarcinoma. The similar results were found in cancer cell lines [26]. Breast cancer cell lines MDA-MB-468, MDA-MB-231, and MCF-7 showed the increased NAMPT expressions compared with the nontransformed MCF-10A cells. Simultaneously, an inverse association between the levels of NAMPT and p73 was reported [27]. In a study of clear cell renal cell carcinoma, NAMPT expression levels were found higher in tumors tissues than in adjacent normal parts [28]. Patients diagnosed with thyroid malignancy were discovered to have higher level of NAMPT expression, and the association of NAMPT level with cancer metastasis and advanced tumor stage was reported [29]. Furthermore, an elevation in NAMPT expression was detected in the experimental myelomatous bones [30]. A comprehensive project to integrate transcriptomic studies from various cancer types with corresponding clinical data was released (University of California, Santa Cruz, *n* = 12,839) [31]. These transcript expression data were obtained by performing RNA-Seq analyses in pancancer; the data retrieved from the TCGA database were normalized and used to assess relative *NAMPT* expression in various types of cancers (Figure 5). *NAMPT* was highly upregulated in glioma, lung cancer, head and neck cancer, and melanoma. In contrast, lower *NAMPT* levels were observed in thyroid cancer, testis cancer, endometrial cancer, and ovarian cancer.

## 5. NAMPT and Clinical Significance

The correlations of the NAMPT level with survival rate in various types of cancers have been addressed and reported. *NAMPT* was discovered to be overexpressed in colorectal cancer, and its expression was associated with inferior overall survival [19]. Similar results were reported in another study showing that overexpressed NAMPT in colorectal cancer tissues was correlated with a poor survival rate [24]. In a clinical study of 261 colorectal cancer patients, high expression of NAMPT determined by immunohistochemical staining appeared to be correlated with advanced TNM stage, vascular invasion, invasion depth, and unfavorable overall survival and disease-free survival time [32]. NAMPT expression was reported to be associated with unfavorable overall survival in melanoma patients [33]. Furthermore, patients of hepatocellular carcinoma exhibiting higher serum NAMPT levels were discovered to have poor overall survival than those with lower serum NAMPT levels (*p* < 0.001) [34]. The results of a Kaplan–Meier analysis of 87 colorectal carcinoma patients indicated a statistically inverse association between NAMPT levels and overall survival rate [35]. A breast cancer study enrolling 176 cancer biopsy tissues indicated that NAMPT is a prognostic biomarker due to the finding its correlation with shorter survival [27]. In addition, the analytical results of a cohort study indicated that the combination of the status of estrogen receptor-negative and high NAMPT levels in serum correlated with the poor disease-free survival in breast cancer [36]. The concurrent status of high NAMPT level and poor survival outcome was characterized in variant types of cancer, including gastric cancer [37], gastric cancer with diabetes [38], upper tract urothelial carcinoma [39], bortezomib-resistant myeloma [40], and breast cancer [41,42]. A comprehensive pancancer *NAMPT* RNA expression profile, determined by microarray and RNA-seq approaches, has been published, and matched patients’ clinical data in the public databases Human Protein Atlas (HPA) [18,43,44,45,46] and Kaplan–Meier plotter [47] are available. The prognostic value of NAMPT in various cancer types is illustrated and shown in Table 2 and Table 3. NAMPT appears to be an inferior prognostic biomarker in cohorts of patients with head and neck cancer, glioma, pancreatic cancer, renal cancer, urothelial cancer, endometrial cancer, and cervical cancer. On the other hand, in patients diagnosed with colorectal cancer and gastric cancer determined by array, those with high NAMPT expression levels are correlated with better clinical outcomes. In clinical studies, we observed discrepancies across different research teams. These inconsistent results might be caused by several variables, including differences in race, quality of care in hospitals, case number per cohort, detection platform (e.g., next-generation sequencing vs. microarray technology), endpoint setting, and random errors.

## 6. NAMPT and Hepatoma

In addition to the direct involvement of NAMPT, the correlation of NAMPT levels with hepatoma growth has been studied. Liver cancer cells surviving hypoxic or oxidative damage showed rapid cell proliferation with increased production of hydrogen sulfide (H2S), which further upregulated NAMPT expression. The inactivation of the NAMPT pathway by FK866 revealed a decrease in H2S and ATP levels [48]. NAMPT appeared to preferentially promote the cell proliferation capability of human hepatoma Hep3B, HepG2, and HuH7 cells as compared with that in normal hepatocytes. This biological effect was reversed by treatment with an inhibitor to inactivate the PI3K-MEK1-GSK3β signaling axis [49]. In addition, NAMPT inhibition by FK866 led to an increase in cell death, AMPKα activation, and repression of mTOR as well as the downstream molecular targets, p70S6 kinase and 4EBP1, in hepatocarcinoma cells. Interestingly, noncancerous hepatocytes showed less sensitivity to FK866 treatment [50]. The cell migration ability of hepatocellular carcinoma was induced by NAMPT in a miR-21-dependent manner [22]. Furthermore, a specific noncompetitive inhibitor for NAMPT, FK866, showed significant induction of delayed cell death in human HepG2 cells, indicating a potentially novel approach for triggering apoptosis in cancer cells [51].

## 7. NAMPT and Breast Cancer

Reduction of NAMPT expression by knockdown led to a significant decrease in cell viability and concentration of NAD in breast cancer MDA-MB-231 cells. Consistently, the apoptosis rate was also increased, according to cell labeling with propidium iodide and annexin V-fluorescein isothiocyanate (FITC) [19]. Inhibition of NAMPT by an FK866 inhibitor led to an increase in sensitivity of triple-negative breast cancers to paclitaxel treatments [52]. In one study, NAMPT appeared to promote breast cancer cell proliferation via the upregulation of Notch1, which resulted in the activation of the NF-κB signaling axis [53]. Furthermore, NAMPT promoted cell proliferation in breast cancer MDA-MB-231 and MCF-7 cells, whereas the effect was abolished by the addition of ERK1/2 and AKT inhibitors [54]. The increased MCF-7 cell proliferation as well as the NAD levels at both extracellular and intracellular parts by NAMPT have also been uncovered, which further indicates that the effect could be reversed by inhibiting enzymatic activity of NAMPT [55]. NAMPT appears to modulate cancer cell angiogenesis. In breast cancer, NAMPT upregulated the expression of the vascular endothelial growth factor (VEGF) genes, matrix metalloproteinase (MMP)-9 and MMP-2, which indicates its potential impacts on angiogenesis [56]. The correlations between clinical pathologic variables and NAMPT levels in serum were reported in breast cancer patients, and the regulation regarding increased MDA-MB-231 cell invasion and migration was uncovered to be via the activation of c-Abl and STAT3 signaling [36]. In contrast, *NAMPT* expression silencing led to a significant induction in metastatic capability as compared with the controls in breast cancer. It was reported that the decrease in NAD^+^ synthesis might result in the aggressiveness in breast cancer MDA-MB-231 cells [57].

## 8. NAMPT and Colorectal Cancer

In a colorectal cancer study, NAMPT knockdown led to reduced cell proliferation via impaired Wnt/β-catenin signaling, and Axin-mediated β-catenin degradation was detected. The biological effect could be reversed by treatment with the enzymatic product of NAMPT, nicotinamide mononucleotide. Conversely, ectopic NAMPT expression increased tumor growth [24]. An increased nicotinamide adenine dinucleotide pool was reported to trigger colon cancer progression by decreasing reactive oxygen species (ROS) levels. The addition of the inhibitor FK866 reduced cancer nodule size by increasing ROS levels in an animal model [58]. NAMPT modulated cell proliferation through the activation of Sirt1-P53 axis. The lentivirus-mediated NAMPT knockdown or NAMPT inhibitor FK866 abolished cancer cell growth and induced cell apoptosis through the upregulation of caspase-3 [59]. The trigger of the epithelial-to-mesenchymal transition by NAMPT was indicated, and the results suggested that NAMPT could induce Snail expression in cancer cells via Akt-GSK-3β-β-catenin axis activation [35]. Emerging experimental results have also indicated the involvement of NAMPT in regulating chemoresistance in cancer. The plasma level of NAMPT appears to be a prognostic indicator for predicting poor response of 5-fluorouracil treatment. The higher NAMPT level was detected in patients experiencing disease progression as comparing with those in the groups of stable disease and partial response [60]. Moreover, the NAMPT level was found to be elevated in established HCT-116 and SW480 cancer cells resistant to doxorubicin. NAMPT knockdown led to an increase in drug sensitivity in cancer cells via the upregulation of multidrug resistance 1 (MDR1) as well as the increased p65 activation determined by nuclear localization [61].

## 9. NAMPT and Melanoma

NAMPT overexpression increased MAPK signaling activation, colony-formation capacity, mesenchymal phenotype, and the drug-effluxing stem cell-like side population (SP) of cells and promoted tumorigenicity in vivo in a highly aggressive type of skin cancer, metastatic melanoma harboring serine-threonine protein kinase B-RAF (BRAF) mutation [62]. In BRAF^V600E^ melanoma cells, the inhibition of NAMPT reduced cancer cell proliferation both in vitro and in vivo, whereas forced NAMPT expression resulted in drug resistance to PLX4032, a potent inhibitor of the BRAF^V600E^ oncogene [63,64]. In melanoma cells, NAMPT induced cancer cell proliferation and abolished p53-mediated apoptosis through the activation of E2F2/SIRT1 signaling pathway [61]. The biological significance of NAMPT in promoting the proliferation rate determined by [^3^H]thymidine incorporation human malignant melanoma Me45 cells as well as in modulating redox adaptative responses by augmenting the activity of antioxidative enzymes, including CAT, GSH-Px, and SOD, were reported [65]. BRAF inhibitor-resistant melanoma cells appeared to have higher NAD levels than cells sensitive to BRAF inhibitors. The treatment of melanoma cells with the NAMPT inhibitor FK866 caused the production of ROS, blocked cancer cells at G2/M phase, leading to cell apoptosis, and improved mouse survival in a xenograft model, demonstrating that NAMPT is a therapeutic target in metastatic melanoma with BRAF mutation [20]. In addition, the role of NAMPT in regulating the tumor microenvironment has been addressed. NAMPT was found to be upregulated in tumor-associated neutrophils from melanoma patients. Targeting NAMPT repressed SIRT1 signaling-mediated neutrophil tumorigenicity, which further abolished the transcription of proangiogenic genes [66].

## 10. NAMPT and Other Cancers

In endometrial cancer, NAMPT was found to promote the cell proliferation in both KLE and Ishikawa cells through the G1/S phase progression triggering by MAPK-ERK1/2 and PI3K-Akt signaling axis activation. In addition, modulation of endometrial carcinoma cell proliferation was also uncovered in a BALB/c-nu mouse model [67]. The NAMPT level is clinically associated with the occurrence of tumor metastasis in various types of cancer. Endometrial cancer patients had statistically higher NAMPT than the controls (*p* = 0.011), and the correlation of NAMPT with deep myometrial invasion (*p* = 0.019) had been reported [68]. Moreover, the NAD biosynthetic axis is required for renal cell carcinoma growth. The NAMPT inhibitor KPT-9274 was reported to inhibit the signaling axis and resulted in a reduction in G2/M transit and an increase of cell apoptosis in specific human renal cell carcinoma cell lines [69]. In addition, pharmacological repression of NAMPT by the inhibitor FK866 reduced glycolytic activity and NAD concentration, which resulted in an increase in antitumor effect of gemcitabine in cell model and orthotopic animal models of pancreatic ductal adenocarcinoma [26]. The combination of radiotherapy and GMX1777 that abolishes NAMPT activity has been in vivo tested in head and neck cancer. The tumor microvessel density determined by CD31 level was significantly decreased to 18% upon GMX1777 addition (*p* < 0.01), and decreased to 4% while combining with radiotherapy (*p* < 0.01) in FaDu cancer cells [70]. Furthermore, the increase of NAMPT in the serum samples from small cell lung cancer patients associated with the brain metastasis [71]. Importantly, NAMPT showed the ability to induce CC chemokine ligand 2-mediated small-cell lung cancer NCI-H446 cell migration in a blood–brain barrier model in vitro [71]. In non-small-cell lung cancer, the NAMPT levels in plasma were uncovered to correlate with the distant metastasis (*p* = 0.003) and the lymph node metastasis (*p* = 0.015). Another lung cancer study indicated that NAMPT could promote migration, invasion, and wound closure, which concurred with the induced increase of MMP-2 and MMP-9 in H358 and A549 cells. The effects were further found to be abolished by the treatment of NF-κB inhibitor, BAY 11-7082 [72]. In addition, NAMPT appeared to reduce doxorubicin sensitivity in non-small-cell lung cancer H1793 and A549 cells through the activation of Akt-ABCC1 signaling pathway. The NAMPT mRNA expression and protein levels appeared to be significantly elevated in non-small-cell lung cancer cells with doxorubicin resistance. NAMPT also promoted the localization of Akt to the ABCC1 promoter region and increased the transcriptional activation [73]. Moreover, the correlations of NAMPT levels in plasma with cancer invasion depth, tumor node metastasis stage, distant and lymph node metastasis were reported in a gastric cancer cohort study [37]. NAMPT revealed the capability to promote the migration and invasion in vitro in osteosarcoma HOS and MG-63 cell. The upregulation of fibronectin and MMP-2 via the activation of NF-κB-IL-6 axis was observed [74]. Moreover, ascites-derived NAMPT was studied regarding its biological function in promoting progression in ovarian cancer. Ascites-derived NAMPT appeared to induce the migration ability in ovarian cancer cells through Rho-ROCK signaling axis to trigger actin stress fiber aggregation, actin polymerization and formation of filopodia and lamellipodia [75]. The NAMPT inhibitor GMX1778 further enhanced the efficacy of 177Lu-DOTATATE treatment for neuroendocrine tumors [76]. A study indicated that repression of NAMPT axis by inhibitors CHS828 and FK866 sensitized the temozolomide treatment via ROS-JNK signaling pathway activation in glioblastoma [77]. The involvement of NAMPT in myeloma progression was reported. The cotreatment of NAD^+^ depletion by FK866, an NAMPT inhibitor, and bortezomib were found to activate caspase 3, caspase 8, caspase 9, and poly (ADP-ribose) polymerase, resulting in a synergistic effect in inducing cell death. *NAMPT* gene silencing also increased the sensitivity to bortezomib in myeloma, indicating NAMPT’s role in drug resistance [40]. Moreover, *NAMPT* gene knockdown by specific siRNA in multiple myeloma RPMI 8226 cells appeared to repress proliferation and induce apoptosis [78].

## 11. NAMPT and Endothelial Cells

Modulation of vascular endothelial cells determines cancer progression, and emerging experimental results suggest that NAMPT plays pivotal roles in this process. NAMPT promote the thromboxane synthase-mediated IL-8 production, which led to the increased angiogenesis in endothelial cells [79]. NAMPT also exerted the angiogenic function by activating mTOR signaling pathway, thereby upregulating hypoxia inducible factor 1α (HIF 1α) and vascular endothelial growth factor (VEGF) in human endothelial cells [80]. In addition, NAMPT appeared to promote tubular formation ability of endothelial cells via the stimulation of Notch1-dependent fibroblast growth factor 2 gene expression [81]. IL-6 was required for the NAMPT-mediated endothelial cell angiogenesis, and the phenotypes determined by rat aortic ring assay, tube formation, and mouse Matrigel plug assay could be further abolished by neutralization of IL-6 activity or inactivation of STAT3 axis [81,82]. Moreover, NAMPT demonstrated the both time- and dosage-dependent biological effects in terms of the increased tubular formation capacity through the activation of PI3K-DDAH2-VEGF signaling axis in human umbilical vein endothelial cells. The aforementioned angiogenic effect cloud be blocked by addition of PI3K inhibitors and dimethylarginine dimethylaminohydrolase 2 (DDAH2) siRNA [83]. The experimental results from another research group further pointed out the MCP-1-CCR2 receptor signaling axis-dependent effect of NAMPT, which significantly promoted capillary tube formation in endothelial cells [84]. The function of triggering capillary-like tube formation was increased by the NAMPT-mediated upregulation of VEGF, MMP-9 and MMP-2 as well as the repression of tissue inhibitors of MMP-1/2 (TIMP-1 and TIMP-2) in a dosage-dependent manner [85]. Importantly, the induction of neovascularization in mouse matrigel plug assay and chick chorioallantoic membrane by NAMPT was reported [86].

## 12. Conclusions 

**Figure 6:**
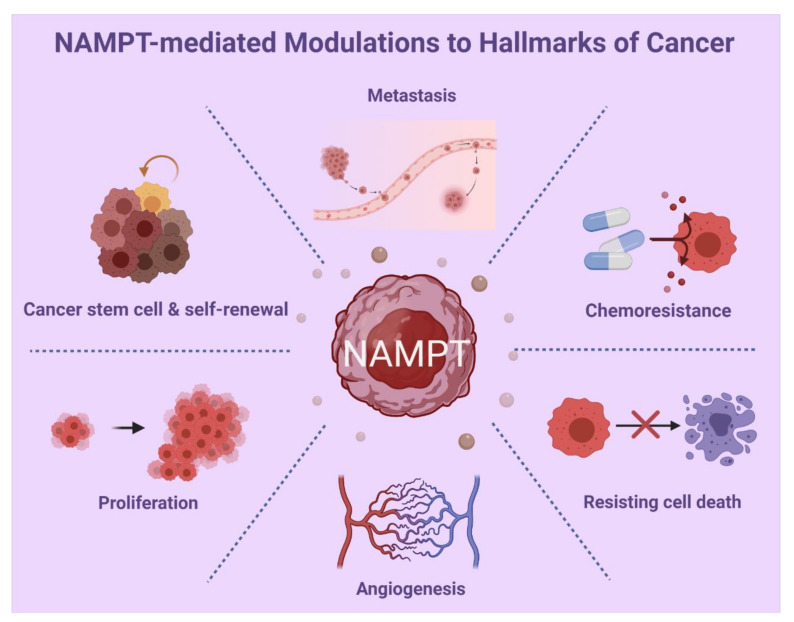
The representative scheme of NAMPT’s modulations to hallmarks of cancer.

Accumulat ed data of in silico analyses from clinical cancer databases and the literature demonstrate *NAMPT* expression in many cancer types. In particular, NAMPT-mediated signaling pathways play a pivotal role in modulating key processes involved in cancer progression, including angiogenesis, cancer cell metastasis, proliferation, cancer stemness, and chemoresistance to anticancer drugs, which are summarized and shown (Figure 6). We presented the relative RNA expression levels of *NAMPT* in a pancancer panel. The observation of differences in RNA expression in specific tumor types indicates the potentially critical regulatory mechanisms that control the activation or repression of *NAMPT* at the level of upstream transcriptional activity. In addition, alterations in determining RNA stability in cancer cells might be of value for further exploration. In the clinic, the discrepancies in prognosis and survival rate across different research teams might be due to differences in factors, including race, case number per cohort, and detection platform. Given the complicated interaction networks involved, these factors may also result in the discrepancies in clinical outcomes. In addition to the differences in experimental procedures used by research groups, NAMPT-mediated biological effects might be partly determined by interactive cofactors and receptors that are still unknown for specific cancer types.

## Figures and Tables

**Table 1:** *NAMPT* mutations in a pancancer study of whole genomes.

Sample ID	Cancer Type Detailed	Protein Change	Mutation Type	Variant Type	Copy Number	Mutations in Sample
#1	Mucinous Adenocarcinoma of the Colon and Rectum	Y69H	Missense	SNP	Diploid	11,036
#2	Mucinous Adenocarcinoma of the Colon and Rectum	L70V	Missense	SNP	Diploid	11,036
#3	Melanoma	G110K	Missense	DNP	Gain	1246
#4	Melanoma	A162T	Missense	SNP	Gain	584
#5	Breast Invasive Ductal Carcinoma	K170E	Missense	SNP	Diploid	81
#6	Prostate Adenocarcinoma	A208T	Missense	SNP	Diploid	10
#7	Colorectal Adenocarcinoma	K229T	Missense	SNP	Diploid	12,010
#8	Melanoma	P238L	Missense	SNP	Gain	2784
#9	Papillary Stomach Adenocarcinoma	K330Q	Missense	SNP	Diploid	59
#10	Esophageal Adenocarcinoma	E366 *	Nonsense	SNP	ShallowDel	242
#11	Renal Clear Cell Carcinoma	G381V	Missense	SNP	Gain	78
#12	Mucinous Adenocarcinoma of the Colon and Rectum	V404A	Missense	SNP	Diploid	11,036

*: nonsense mutation.

**Table 2:** Correlation of *NAMPT* with cancer patient survival.

Symbol	Cancer Type	Prognosis	Endpoint	*p* Value	Case	Dataset	Method
*NAMPT*	Glioma	Poor	Overall survival	0.014	153	TCGA	RNA Seq
*NAMPT*	Thyroid Cancer	-	Overall survival	N.S.	501	TCGA	RNA Seq
*NAMPT*	Lung Cancer	-	Overall survival	0.027	994	TCGA	RNA Seq
*NAMPT*	Colorectal Cancer	Good	Overall survival	0.0059	597	TCGA	RNA Seq
*NAMPT*	Head and Neck Cancer	Poor	Overall survival	<0.001	499	TCGA	RNA Seq
*NAMPT*	Stomach Cancer	-	Overall survival	N.S.	354	TCGA	RNA Seq
*NAMPT*	Liver Cancer	-	Overall survival	N.S.	365	TCGA	RNA Seq
*NAMPT*	Pancreatic Cancer	Poor	Overall survival	<0.001	176	TCGA	RNA Seq
*NAMPT*	Renal Cancer	Poor	Overall survival	<0.001	877	TCGA	RNA Seq
*NAMPT*	Urothelial Cancer	Poor	Overall survival	0.0064	406	TCGA	RNA Seq
*NAMPT*	Prostate Cancer	-	Overall survival	N.S.	494	TCGA	RNA Seq
*NAMPT*	Testis Cancer	-	Overall survival	N.S.	134	TCGA	RNA Seq
*NAMPT*	Breast Cancer	-	Overall survival	N.S.	1075	TCGA	RNA Seq
*NAMPT*	Cervical Cancer	Poor	Overall survival	<0.001	291	TCGA	RNA Seq
*NAMPT*	Endometrial Cancer	Poor	Overall survival	0.016	541	TCGA	RNA Seq
*NAMPT*	Ovarian Cancer	-	Overall survival	N.S.	373	TCGA	RNA Seq
*NAMPT*	Melanoma	-	Overall survival	N.S.	102	TCGA	RNA Seq

Survival data were collected from the Human Protein Atlas and TCGA databases. N.S.: no significance.

**Table 3:** Correlation of *NAMPT* with cancer patient survival.

Symbol	Cancer Type	Prognosis	Endpoint	*p* Value	Case	Dataset	Method	Probe ID
*NAMPT*	Breast Cancer	-	Relapse-free survival	N.S.	2032	E-MTAB-365, E-TABM-43, GSE: 11,121, 12,093,	Array	1555167_s_at
						12,276, 1456, 16,391, 16,446, 16,716, 17,705, 17,907,		
						18,728, 19,615, 20,194, 20,271, 2034, 20,685, 20,711,		
						21,653, 22,093, 25,066, 2603, 26,971, 29,044, 2990,		
						31,448, 31,519, 32,646, 3494, 36,771, 37,946, 41,998,		
						42,568, 43,358, 43,365, 45,255, 4611, 46,184, 48,390,		
						50,948, 5327, 58,812, 61,304, 65,194, 6532, 69,031,		
						7390, 76,275, 78,958, 9195		
*NAMPT*	Ovarian Cancer	-	Progression-free survival	N.S.	614	GSE: 14,764, 15,622, 18,520, 19,829, 23,554, 26,193,	Array	1555167_s_at
						26,712, 27,651, 30,161, 3149, 51,373, 63,885, 65,986,	RNA Seq	
						9891, TCGA (*n* = 565)		
*NAMPT*	Lung Cancer	-	Post-progression survival	N.S.	138	CAARRAY, GSE: 14,814, 19,188, 29,013, 30219,	Array	1555167_s_at
						31,210, 3141, 31,908, 37,745, 43,580, 4573, 50,081,	RNA Seq	
						8894, TCGA (*n* = 133)		
*NAMPT*	Gastric Cancer	Good	Post-progression survival	0.0024	384	GSE: 14,210, 15,459, 22,377, 29,272, 51,105, 62,254	Array	1555167_s_at

Survival data were collected from the Human Protein Atlas and TCGA databases. N.S.: no significance.
